# Genetic screening in sudden cardiac death in the young can save future lives

**DOI:** 10.1007/s00414-015-1237-8

**Published:** 2015-07-31

**Authors:** Eva-Lena Stattin, Ida Maria Westin, Kristina Cederquist, Jenni Jonasson, Björn-Anders Jonsson, Stellan Mörner, Anna Norberg, Peter Krantz, Aase Wisten

**Affiliations:** 1 Department of Medical Biosciences, Medical and Clinical Genetics, Umeå University, Umeå, Sweden; 2 Heart Centre and Department of Public Health and Clinical Medicine, Umeå University, Umeå, Sweden; 3 Department of Forensic Medicine, Lund University, Lund, Sweden; 4 Department of Internal Medicine, Sunderby Hospital, Luleå, Sweden

**Keywords:** Sudden unexplained death, Sudden cardiac death, Molecular autopsy, Long QT syndrome, Catecholaminergic polymorphic ventricular tachycardia

## Abstract

**Background:**

Autopsy of sudden cardiac death (SCD) in the young shows a structurally and histologically normal heart in about one third of cases. Sudden death in these cases is believed to be attributed in a high percentage to inherited arrhythmogenic diseases. The purpose of this study was to investigate the value of performing post-mortem genetic analysis for autopsy-negative sudden unexplained death (SUD) in 1 to 35 year olds.

**Methods and results:**

From January 2009 to December 2011, samples from 15 cases suffering SUD were referred to the Department of Clinical Genetics, Umeå University Hospital, Sweden, for molecular genetic evaluation. PCR and bidirectional Sanger sequencing of genes important for long QT syndrome (LQTS), short QT syndrome (SQTS), Brugada syndrome type 1 (BrS1), and catecholaminergic polymorphic ventricular tachycardia (CPVT) (*KCNQ1*, *KCNH2*, *SCN5A*, *KCNE1*, *KCNE2*, and *RYR2*) was performed. Multiplex ligation-dependent probe amplification (MLPA) was used to detect large deletions or duplications in the LQTS genes. Six pathogenic sequence variants (four LQTS and two CPVT) were discovered in 15 SUD cases (40 %). Ten first-degree family members were found to be mutation carriers (seven LQTS and three CPVT).

**Conclusion:**

Cardiac ion channel genetic testing in autopsy-negative sudden death victims has a high diagnostic yield, with identification of the disease in 40 % of families. First-degree family members should be offered predictive testing, clinical evaluation, and treatment with the ultimate goal to prevent sudden death.

## Introduction

Sudden cardiac death (SCD) in the young is a tragic event, with an incidence of 1–2 per 100,000 per year, in the age group 15 to 35 years old [1–4]. SCD is defined as a witnessed, natural unexpected death from cardiac causes occurring within 1 h after onset of symptoms in a previously healthy person or an unwitnessed natural unexpected death of a person observed to be well within 24 h of being found dead [5]. Standard forensic autopsy is often unsuccessful in determining the causes of sudden death [1, 6–8]. Negative toxicology and a structurally and histologically normal heart is found in up to 53 % of these cases, commonly referred to as autopsy-negative, sudden unexplained death (SUD) [1, 3, 9]. There are several conditions difficult to discover at autopsy that may cause SUD. Inherited cardiac diseases such as channelopathies and cardiomyopathies are a plausible cause of such autopsy-negative SCD cases and may explain a significant number of these events [10–13]. Studies have shown that genetic testing may identify a cardiovascular cause of death in up to 30 % of these cases [11, 14–19]. Long QT syndrome (LQTS), short QT syndrome (SQTS), Brugada syndrome (BrS), and catecholaminergic polymorphic ventricular tachycardia (CPVT) causing isolated electric dysfunction are due to defective genes encoding subunits of cardiac ion channels (K^+^, Na^+^, or Ca^2+^) or ion channel regulatory proteins [20, 21]. Disturbed ion flow is the substrate to ventricular arrhythmias causing syncope and increased risk of SCD, which characterize these disorders. Syncope is the most common symptom in ion channel disorders, although cardiac arrest may be the first clinical manifestation in apparently healthy subjects. In LQTS, death is the first symptom of the disease in 10–15 % of cases, [21] whereas in CPVT, death can be the first clinical presentation in up to 30 % of cases [22]. The mean penetrance of pathogenic *RYR2* variants causing CPVT is 83 %, with mortality rates of 30–50 % by age 35 years, and consequently, asymptomatic carriers are rare [20, 22]. It is also useful to test for cardiomyopathies such as hypertrophic cardiomyopathy (HCM) and arrhythmogenic right ventricular cardiomyopathy (ARVC) as structural changes in the cardiac tissue connected with these diseases can be difficult to discover at autopsy [17]. In this nationwide prospective study, our first aim was to determine the value of molecular genetic autopsy in young SUD victims by genetic screening of genes important for LQTS, SQTS, BrS1, and CPVT. Mutation carriers are often unaware of their disease, and unfortunately, sudden death in many cases is the first chance to reveal the disorder. Molecular autopsy can give an explanation to the sudden death, which is important for the mourning process of the family [23]. The findings of an inherited genetic disorder also provide an opportunity to offer first-degree family members cascade screening; identification of mutation carriers can save future lives [16, 24, 25]. Our second aim was to offer first-degree relatives genetic testing and medical surveillance to mutation carriers.

## Methods

### Inclusion criteria

Between January 2009 and December 2011, the Swedish departments of forensic medicine were invited to send whole blood in EDTA tubes and tissue samples from cases of SUD. Toxicological analysis including illicit drugs and narcotics was performed in all cases. Histological examination of the heart were performed, comprising both the left and right ventricle, and in some cases also the conduction system, the average number of samples taken from the heart was 11 with a range from 3 (one case) to 26. The specimens were examined by trained investigators in haematoxilin-eosin staining as well as PTAH staining. To be included in the study, the deceased had to be 1–35 years old and the death had to be sudden, unexpected, and unexplained after comprehensive medicolegal autopsy. Unnatural causes of death such as trauma, murder, suicide, or intoxication were excluded. In total, 15 SUD victims (10 male and 5 female, medium age at death 15 years) fulfilled these criteria and were included in the study.

### Clinical data

Data on included SUD victims were collected from forensic autopsy reports and police reports. Information on preceding symptoms and medical history of the deceased was assembled from these sources supplemented with interviews with first-degree relatives and medical records when available.

### Genetic analyses

The genetic analyses and interpretation of pathogenicity of sequence variants have been performed as part of ordinary health care diagnostics at a clinical genetics laboratory experienced with sequence analysis of ion channel disorders. DNA was extracted from blood or tissue samples using standard salting-out methods. DNA were analyzed for sequence variants in all protein-coding exons and their flanking splice site regions of the genes *KCNQ1* (NM_000218.2 and NM_181798.1), *KCNH2* (NM_000238.2 and NM_172057.1), *SCN5A* (NM_198056.1), *KCNE1* (NM_000219.2), and *KCNE2* (NM_172201.1) using PCR and bidirectional sequencing on an ABI3500 Genetic Analyzer (Applied Biosystems, Foster City, CA, USA). DNA were also analyzed for sequence variants in the functionally most important exons (8–15, 44–50, 83, 88–105) of the gene *RYR2* (NM_001035.2). Two cases with pathogenic sequence variants in the *KCNQ1* gene were only screened for sequence variants in the *KCNQ1* gene. Primers were M13-tagged and modified from Jongbloed et al. or designed with Primer3 software (primer sequences are available upon request). To detect deletions or duplications, five samples were analyzed using multiplex ligation-dependent probe amplification (MLPA) and the SALSA P114-A2 kit (MRC-Holland). This kit covers exons 1B, 1–4, 6–13, and 15–16 of *KCNQ1*, exons 1B, 1–4, 6, 9–10, and 14 of *KCNH2*, exons 1–2 of *KCNE2*, exons 2–4 of *KCNE1*, and exons 2, 4, 25, and 27 of *SCN5A*. Direct DNA sequencing of the protein coding exon was used for cascade screening of family members.

### Interpretation of identified sequence variants

Sequences were evaluated using the Sequencer software version 4.9 (Gene Codes Corporation, MI, USA) and compared with a reference genomic sequence. All identified variants were denoted using accepted nomenclature recommended by the Human Genome Variation Society. In silico predictions were made for all identified sequence variants using the Alamut software version 2.2 (Interactive Biosoftware, Rouen, France). The Alamut software assists in evaluation of missense variants by compiling output from a number of bioinformatic prediction tools, including PolyPhen, SIFT, Align-GVGD, and MutationTaster [26–28]. Novel missense variants were tested for presence in 200 control chromosomes of the same population and checked for presence in the 6500 control exomes of the NHLBI Exome sequencing project.

### Ethics approval

The Regional Ethical Review Board in Umeå, Sweden, approved this study. All first-degree relatives have given their written informed consent.

## Results

Fifteen SUD victims aged 1–35 years with a structurally normal heart at autopsy were referred for post-mortem ion channel genetic testing, during the period 2009–2011. We estimate that this represent approximately 50 % of SUD cases in this period, according to ongoing investigation of SCD among the young in Sweden 2000–2010 (unpublished data). Toxicological analysis including illicit drugs and narcotics were negative with exception for two cases with therapeutic concentrations of relatively harmless drugs. All cases had normal macroscopic hearts, with the exception of two obese individuals (BMI 33 and 35) who had mild enlargement of their hearts. Histological examination of the left and right ventricle of the heart and in some cases also the conduction system showed no pathological changes that could explain the deaths. Clinical characteristics of the SUD cases are summarized in Table 1. The mean age of death of the SUD victims was 15.5 ± 13 years (mean ± SD), range 2–28 years. Two of the SUD victims had a family history of sudden death at the time of death. The majority of the subjects, 9/15 (60 %) died during rest or sleep. Two subjects (13 %) died during physical activity: one after hurrying to the bus stop and one drowned. Two subjects died during daily activity: one sitting in his classroom at school and one died in a car accident. Two subjects died at home without anyone knowing details of the event (Table 1).

**Table 1:** Clinical history, autopsy finding, and molecular genetic evaluation of 15 sudden unexplained death victims in Sweden 2009–2011, 1–35 years of age

ID	Age	Sex	Activity	Details of event	Clinical history	Investigation	Family history	Autopsy findings	Genetic findings	Familial cascade screening	Diagnosis
1	28	F	Unknown	Found deceased at home Not witnessed	Syncope 2 days before death Medication: mirtazapine	No ECG Clinical examination	Unknown	Inflammatory changes in the heart	KCNQ1 p. Q220K	1 neg (adopted)	LQTS
2	8	F	Sleep	Found deceased at home Not witnessed Infection	Healthy	None	None	Normal heart	KCNQ1 p. H240R	3 pos/1 neg	LQTS
3	18	M	Unknown	Found deceased on the floor at home Not witnessed	Had a drinking problem Medication: alprazolam, alimemazine. Had a cold 2 weeks before death	None	None	Normal heart. Ethanol in femoral blood (1.06‰), alprazolam, alimemazine in therapeutic concentrations	KCNQ1 p. A370V	4 pos/3 neg	LQTS
4	14	M	Sitting	Collapsed in school Witnessed	ADHD medication: tomoxetine	None	Unknown	Normal heart	KCNH2 p. S668L	None (adopted)	LQTS
5	18	M	Physical activity	Collapsed at the bus stop Witnessed	Cardiac arrest 3 years before death High blood pressure Fever spells in childhood and suspected seizures as teenager	ECG, cardiac ultra sound and EEG normal	Sister, grand grandfather and grand uncle SUD	Normal heart	RYR2 p. S4168T	3 pos/3 neg	CPVT
6	14	M	Physical activity	Drowning Not witnessed	Healthy	None	None	Normal heart	RYR2 p. D401G De novo	3 neg	CPVT
7	18	M	Sleep	Found deceased at home Not witnessed	Tachycardia 1½ years before death	None No ECG	None	Normal heart	None	–	Causa mortis ignota
8	17	M	Sleep/rest	Found deceased at home Not witnessed	Seizure 1 week before death Had a cold 3 weeks before death	No ECG	None	Normal heart	None	–	Causa mortis ignota
9	2	M	Sleep	Found deceased at home Not witnessed	1 year before death sudden un-consciousness and falling from a bobby car	None	None	Normal heart	None	–	Causa mortis ignota
10	3	M	Sleep	Found deceased at home Not witnessed	Infection the day before death, fever seizures earlier	None	Father seizure, grandfather SUD	Normal heart	None	–	Causa mortis ignota
11	18	M	Sleep	Found deceased at home Not witnessed	Vomiting and palpitations some days before death	None	None	Normal heart	None	–	Causa mortis ignota
12	17	F	Sleep/rest	Found deceased at home Not witnessed	Vomiting, headache, and dizziness during the night before death	None	None	Normal heart	None	–	Causa mortis ignota
13	18	M	Sleep	Found deceased at home Not witnessed	The night before death vomiting, and hard physical activity	None	None	Normal heart In urine: metandienon; 945 ng/ul	None	–	Causa mortis ignota
14	17	M	Sleep	Found deceased at home Not witnessed	Diabetes	None	None	Normal heart, glucose, and lactate normal concentrations	None	–	Causa mortis ignota
15	22	F	Driving	Car accident Included because no injury that could explain the death	Pregnant	None	None	Normal heart	None	–	Causa mortis ignota

*ECG* electrocardiogram, *LQTS* long QT syndrome, *ADHD* attention deficit hyperactivity disorder, *EEG* electroencephalogram, *SUD* sudden unexpected death, *CPVT* catecholaminergic polymorphic ventricular tachycardia, *Causa mortis ignota* death cause unknown

### Genetic testing of ion channel genes and clinical description of cases

Genetic screening of the *KCNQ1*, *KCNH2*, *SCN5A*, *KCNE1*, *KCNE2*, and *RYR2* genes revealed four LQTS and two CPVT mutations (40 %). There were no deletions or duplications detected with MLPA. None of the pathogenic sequence variants had previously been reported in the literature, dbSNP, or 1000 Genomes, and none were identified in the NHLBI Exome Sequencing Project database (ESP), where >6000 European-American and African-American individuals were available. All of the novel variants were predicted to be pathogenic according to at least two bioinformatic tools [26–28]. Characteristics of the identified pathogenic sequence variants are given in Table 2.

**Table 2:** Characteristics of pathogenic sequence variants found in 15 victims of sudden unexplained death aged 1–35 years in Sweden 2009–2011

Gene	Exon	Nucleotide change	Amino acid change	Region	Domain	Polyphen-2^a^	SIFT	MutationTaster^b^	Align-GVGD^c^
*KCNQ1*	4	c.658C>A	p. Gly220Lys	S3-S4	Ion transport	Possibly damaging	Tolerated	Disease causing	C0
	5	c.719A>G	p. His240Arg	S4	Ion transport	Benign	Deleterious	Disease causing	C25
	8	c.1109C>T	p. Ala370Val	C-term	–	Probably damaging	Deleterious	Disease causing	C65
*KCNH2*	8	c.2003C>T	p. Ser668Leu	S6		Probably damaging	Deleterious	Disease causing	C65
*RYR2*	14	c.1202A>G	p. Asp401Gly	N-term	MIR motif	Possibly damaging	Deleterious	Disease causing	C65
	90	c.12502T>A	p. Ser4168Thr	Cytosol	–	Possibly damaging	Deleterious	Disease causing	C55

*SIFT* sorting intolerant from tolerant

^a^Polyphen-2, polymorphism phenotyping v2, predicts possible impact of an amino acid substitution on the structure and function of the protein

^b^MutationTaster calculates probability for the alteration to be either disease causing or a harmless polymorphism

^c^*Align-GVGD* Align Grantham variation and Grantham distance, ordered from most likely (C65) to interfere with function to least likely (C0)

### Identified LQTS mutations

#### Individual 1

A pathogenic sequence variant in the *KCNQ1* gene (Gly220Lys) was identified in a 28-year-old adopted female who was found dead at home during daytime. The woman had given birth to a child 9 months prior to her death. She was depressed, had disturbed sleeping pattern, and was treated with benzodiazepines. She had experienced syncope 2 days prior to the death. The physician at the hospital judged the episode as a vasovagal syncope, and no ECG was taken. The child did not carry the pathogenic sequence variant, and no other family members were available for genetic testing.

#### Individual 2

A pathogenic sequence variant in the *KCNQ1* gene (His240Arg) was identified in an 8-year-old girl who had a cold and died during sleep. Predictive testing of family members revealed three carriers of the pathogenic sequence variant. The mother of the deceased child was found to be a carrier; she was adopted, and nothing is known about her biological parents/siblings. Two siblings were also carriers of the pathogenic sequence variant; one of them has a prolonged QT duration; but none of them have experienced syncope.

#### Individual 3

A pathogenic sequence variant in the *KCNQ1* gene (Ala370Val) was identified in an 18-year-old man who was found dead on the floor at home. Toxicology examinations showed alimemazine and alprazolam in normal therapeutic concentrations in his blood and 1.06‰ ethanol in femoral blood. Cascade screening of family members identified four mutation carriers and three none carriers. Two of the carriers have experienced symptoms possible due to LQTS: his grandmother has felt dizziness while swimming a couple of times, and his uncle has experienced dizziness and pre-syncope.

#### Individual 4

A pathogenic sequence variant in the *KCNH2* gene (Ser668Leu) was identified in a 14-year-old boy who died sitting at his desk in school. Cardiopulmonary resuscitation was started within minutes but was unsuccessful. The boy was treated with tomoxetine due to attention deficit hyperactivity disorder (ADHD). The boy was adopted; therefore, no genetic investigation of parents or siblings was possible.

### Identified CPVT mutations

#### Individual 5

A pathogenic sequence variant in the *RYR2* gene (Ser4168Thr) was identified in an 18-year-old man, who collapsed having hurried to the bus stop. Resuscitation was unsuccessful. The man had been resuscitated 3 years earlier after a similar event and thoroughly investigated without any pathological cardiac findings. He had no current symptoms proceeding death and no treatment. Four weeks after his death, prior to the molecular genetic diagnosis, his younger sister died suddenly when walking back to school after a swimming lesson. She was later found to be a mutation carrier. The sister was born with a tetralogy of Fallot; she had recurrent symptoms of palpitations and syncope. Genetic testing showed that their mother and maternal grandfather also carried the mutation, although neither of them had experienced any symptoms. The maternal great grandfather had experienced syncope and died suddenly unexpected at the age of 46 years, and a maternal grand uncle died suddenly at the age of 64 years; however, we do not know their carrier status. Three family members tested negative for the familial mutation.

#### Individual 6

A pathogenic sequence variant in the *RYR2* gene (Asp401Gly) was identified in a 14-year-old boy who drowned, despite that he was a good swimmer. The pathogenic sequence variant was found to be de novo since none of the family members carried the mutation.

In nine of the 15 evaluated cases, we did not identify a disease causing sequence variant; five of them had experienced pre-mortal symptoms, and one had a family history of SUD (Table 1).

## Discussion

This is to our knowledge the first prospective nationwide molecular genetic screening study of the LQTS, SQTS, BrS1, and CVPT genes in SUD victims. Genetic screening of cardiac-channel genes revealed six pathogenic sequence variants (40 %) among 15 afflicted SUD victims, four LQTS mutations, and two CPVT mutations; ten first-degree family members were found to be carriers and 11 noncarriers. None of the identified mutations had previously been described; thus, all of them were unique to this Swedish cohort. In published LQTS population surveys, most of the mutations are novel (≈60 %) [29–31]. We have recently described the LQTS mutation panorama in Sweden, and almost one third (28 %) of the identified mutations were novel [32].

### Comparison to other studies

Skinner et al. evaluated the LQT genes in the first prospective population-based series of SUD victims and identified five pathogenic sequence variants in 33 individuals (15 %) [14]. The diagnostic yield concurs with the result in a Danish study but is lower than in our present study, and in the retrospective autopsy studies from the Mayo clinic and from New Zealand [11, 15, 33]. In a Danish cohort of SUD cases, pathogenic sequence variants was identified in five of 44 (11 %); cases were tested for sequence variants in the LQT1–3 genes [15]. Tester et al. showed in a retrospective autopsy study that one third (17/49) of young SUD cases had verified genetic causes: LQTS (20 %) or CPVT (15 %) [11]. Gladding et al. used DNA from Guthrie cards collected for newborn metabolic screening and identified disease-causing sequence variants in four of 19 SUD cases (21 %) [33]. The high yield in our present study may be due to sequencing also of the *RYR2* gene, ascertainment bias, or a low number of cases. Recently, Bagnall et al. used exome sequencing of 27 genes and identified nine rare variants in a subset of 28 SUD cases (32 %) [34]. Next generation sequencing and whole exome sequencing will likely be a more effective strategy to uncover the etiology of SUD, although there are limitations and disadvantages of such approaches, including the identification of variants of unknown significance and “co-incidental” genetic pathogenic sequence variants that predispose to adult-onset potentially incurable conditions.

### Pre-mortal symptoms are common

In this study, a man with CPVT had been resuscitated after cardiac arrest 3 years before death, and a woman with LQTS experienced syncope 2 days before she died. The other genotype-positive SUD cases had no reported cardiac-related pre-mortal symptoms. In nine individuals, we did not identify pathogenic sequence variants coding for ion channel disorders; five of these had experienced pre-mortal symptoms such as palpitations or seizures. Medical care was seeked by three of them, but no ECGs were taken. In a Swedish SCD cohort of 15–35 year olds between 1992 and 1999, as many as 46 % had requested medical advice because of symptoms before suffering from SCD [35]. It is possible to identify patients with syncope requiring further evaluation for cardiac etiology [36]. Tretter et al. evaluated syncope in a pediatric setting: using the characteristics in the medical history, physical examination, and ECG, to identify all patients with cardiac etiology [36]. There could be cardiac explanations also to the SUD cases in our study, such as cardiomyopathies with minor visible changes, inflammatory injury of the electrical system in the heart, or pathogenic sequence variants that we were not able to identify. The SUD cases could also have undetected noncardiac background such as epilepsy or drug-related causes.

### Activity at time of death

Syncope usually occurs during exercise or acute emotions, due to torsade de pointes (TdP) in LQTS and due to fast ventricular tachycardia in CPVT [21, 22]. Most often, there is a spontaneous recovery of bidirectional or polymorphic ventricular tachycardia and TdP. However, the arrhythmia may degenerate into ventricular fibrillation, subsequently leading to sudden death if no resuscitation is available [21, 22]. In this study, two out of six carriers of a pathogenic sequence variant died during exercise: one while running (LQTS) and one while swimming (CPVT). Increased risk for death while swimming is recognized both in CPVT and LQTS (especially the LQT1 subtype) [21, 37]. In a post-mortem series of drowning victims, pathogenic sequence variants in the *RYR2* gene were identified in 20 % [37, 38]. The majority in our study died during normal daily activity or sleep, which is in accordance with other studies [25].

### Certain drugs prolong the QT duration

It is well known that certain drugs prolong the QT duration and might induce TdP in individuals with LQTS [39]. In this study, three of four SUD cases with an identified pathogenic LQT variant were on medication, thus revealing therapeutic levels of medicine in the blood toxicology screening. Both the drugs alimemazine and tomoxetine are known to prolong the QT duration, although the risk for serious cardiovascular adverse events, including significant increases in QTc and sudden cardiac death, is judged to be low. However, great caution is advised when considering stimulant and nonstimulant medications for patients of any age with a diagnosis of ADHD and a personal or family history or other known risk factors for cardiovascular disease [40]. We do not know if the treatment was of significance for the death of the SUD victims in this study, but we know that the medication was given without knowing the vulnerability due to an at that time undiscovered disease.

### Autopsy for crime investigation

Post-mortem DNA analysis of SUD victims opens new diagnostic possibilities but is to our knowledge not yet included in routine investigation in any country. This is probably because these cases often are handled in the juridical/legal system; it is in some countries not legal to do these examinations without permission of the next kin. The main purpose of the forensic autopsy is to investigate possible crimes and only secondary to investigate cause of death due to disease. Furthermore, the analyses have so far been very expensive, especially in the perspective that the investigations are not carried out as part of a medical mission. It is recommended that all SUD victims undergo expert cardiac pathology to rule out a structural heart disease [41]. Collection of blood in an EDTA tube or a blood spot card or a frozen sample of tissue is recommended in cases of SUD and sudden unexpected death in infancy for subsequent DNA analysis/genetic testing [41].

### Clinical implication

If post-mortem genetic testing was to be performed routinely, these autopsy-negative cases could get a diagnosis, first-degree relatives could have an explanation to the death, and family members could be offered predictive testing. Treatment and surveillance of genotype-positive individuals can reduce morbidity and mortality, and noncarriers would benefit from certain knowledge of not being at risk.

### Limits of the study

Not all genes or exons involved in cardiac ion channel disorder were analyzed. Only about 50 % of the SUD victims were referred for genetic testing. The relatively low participation rate may be due to not considering the molecular diagnosis option immediately at autopsy, but first after all other diagnostic options have failed.

## Conclusions

Our study shows that cardiac-channel focused investigation is of great value in establishing the cause of death in unexplained sudden death, thus supporting the transition of a molecular genetic autopsy into routine clinical practice. In view of what is stated above concerning the primary objective of the investigation of these deaths, it is necessary to establish a partnership between health care and justice; both to implement adequate sampling but also to finance analyses. Furthermore, issues relating to handling of positive test results and privacy problems have to be solved.

## Abbreviations

BrS: Brugada syndrome
CPVT: Catecholaminergic polymorphic ventricular tachycardia
LQTS: Long QT syndrome
MLPA: Multiplex ligation-dependent probe amplification
SCD: Sudden cardiac death
SUD: Sudden unexplained death
SQTS: Short QT syndrome
